# Legal Notes for Institutional Workers

**Published:** 1915-07-17

**Authors:** 


### LEGAL NOTES FOR INSTITUTIONAL WORKERS.

### A Panel Doctor's Fees: Judgment on Appeal.

At the outset this month we may briefly note that the Court of Appeal has affirmed the decision of Mr. Justice Rowlatt—which, at the time it was given, was commented on here at length—to the effect that the fees of a panel doctor received by an Insurance Committee from the Insurance Commissioners are attachable in the hands of the committee by a creditor of the doctor who has done the work.

It is a matter of common procedure for the Court to appoint an officer to take charge of the affairs of an incapacitated person, and doctors are familiar with the certificates which are required for such a purpose. Usually the incapacity in question is mental, but in a recent case we notice that the averments made were simply that the person whose affairs were needing a custodier was almost completely blind and deaf, and these were supported by medical certificates to that effect. The Court was satisfied that protection was necessary, and appointed a *curator bonis* as in the case of an *incapax*.

### Accident to a Hospital Patient.

One of the provisions of the Public Health (Scotland) Act, 1897, is: "Every action or prosecution against any person acting under this Act on account of any wrong done in or by any action, proceeding or operation under this Act shall be commenced within two months after the cause of action shall have arisen." In *Duncan* v. *Magistrates of Hamilton*, 1902, 5 F. 160, a child who, under the powers conferred by the Public Health Act, had been removed by a local authority to the burgh fever hospital, was injured by upsetting over himself a bottle of acid left within his reach by a servant in the course of cleaning the ward. Ten months after the accident an action, claiming damages for personal injury to the child, was brought against the local authority, but was held barred by the time limitation in the statute.

### A Hospital Employee's Negligence.

Now, in *Baker* v. *The Corporation of Glasgow*, 1915, 2 S.L.T. 45, a different result has been reached in the following circumstances. The pursuer claimed damages for personal injuries received by him by his being knocked down by a motor-car belonging to the defenders. The car was, in fact, one of the ambulances attached to a fever hospital maintained by the defendant Corporation under the Public Health Act, and was at the time of the accident being driven by one of the hospital staff, who was solely under the control of the superintendent of the hospital, his wages and all the other expenses incident to the running of the car being paid out of the public health assessment. Also at the time of the accident the car was doing its proper ambulance work—namely, conveying a patient to hospital. The action was raised more than two months after the date of the accident, and the Corporation pleaded that it could not then be insisted in; but the Court repelled that plea and allowed the action to proceed, notwithstanding the lapse of time.

The action could not, it was pointed out, be said to be an action against the public health authority for negligence in the performance of statutory duty. So far as the pursuer was concerned, the action in which the defenders' servant was engaged when the accident occurred was confined entirely to the running of him down, and that was not an action under the statute. The defenders had absolutely no title or right *qua* public health authority or otherwise to interfere in any way with the pursuer. They were not *quoad* him engaged in any action, proceeding or operation under the statute, and could not therefore do him a wrong in the statutory sense. It was a mere accident so far as he was concerned, and it in no way affected his common-law rights of action that when defendants' car struck him they happened to be exercising their statutory powers with reference to someone else.

